# Building the European Antimicrobial Resistance Surveillance network in veterinary medicine (EARS-Vet)

**DOI:** 10.2807/1560-7917.ES.2021.26.4.2001359

**Published:** 2021-01-28

**Authors:** Rodolphe Mader, Peter Damborg, Jean-Philippe Amat, Björn Bengtsson, Clémence Bourély, Els M Broens, Luca Busani, Paloma Crespo-Robledo, Maria-Eleni Filippitzi, William Fitzgerald, Heike Kaspar, Cristina Muñoz Madero, Madelaine Norström, Suvi Nykäsenoja, Karl Pedersen, Lucie Pokludova, Anne Margrete Urdahl, Alkiviadis Vatopoulos, Christos Zafeiridis, Jean-Yves Madec, on behalf of EU-JAMRAI

**Affiliations:** 1 University of Lyon, French Agency for Food, Environmental and Occupational Health and Safety (ANSES), Laboratory of Lyon, Antibiotic Resistance and Bacterial Virulence Unit, 31 avenue Tony Garnier, 69007 Lyon, France; 2 University of Copenhagen, Department of Veterinary and Animal Sciences, Stigbøjlen 4, 1870 Frederiksberg C, Denmark; 3 University of Lyon, French Agency for Food, Environmental and Occupational Health and Safety (ANSES), Laboratory of Lyon, Epidemiology and Support to Surveillance Unit, 31 avenue Tony Garnier, 69007 Lyon, France; 4 National Veterinary Institute, Department of Animal Health and Antimicrobial Strategies, Ulls väg 2B, SE-751 89 Uppsala, Sweden; 5 Direction générale de l’alimentation, Bureau de la santé animale, 75015, Paris, France; 6 Utrecht University, Faculty of Veterinary Medicine, Department of Biomolecular Health Sciences, Yalelaan 1, 3584 CL, Utrecht, the Netherlands; 7 Istituto Superiore di Sanità, Department of Infectious Diseases, Viale Regina Elena 299, 00161 Rome, Italy; 8 Agencia Española del Medicamento y Productos Sanitarios (AEMPS), Coordinación del Plan Nacional Antibióticos (PRAN), calle Campezo 1, EDF. 8. 28022 Madrid, España; 9 Sciensano, Veterinary Epidemiology Unit, Department of Epidemiology and Public Health, Belgian Federal Research Institute for Public and Animal Health, Rue Juliette Wytsmanstraat 14, 1050 Brussels, Belgium; 10 Limerick Regional Veterinary Laboratory, Department of Agriculture, Food and the Marine, Knockalisheen, Limerick, Ireland V94 WK44; 11 Federal Office of Consumer Protection and Food Safety, Mauerstrasse 39-42, 10117 Berlin, Germany; 12 Norwegian Veterinary Institute (NVI), Pb 750 Sentrum, N0106 Oslo, Norway; 13 Finnish Food Authority, Laboratory and Research Division, Microbiology Unit, Mustialankatu 3, 00790 Helsinki, Finland; 14 Institute for State Control of Veterinary Biologicals and Medicines (ISCVBM), Hudcova 56 A, Brno, the Czech Republic; 15 University of West Attica, Department of Public Health Policy, School of Public Health, Athens, Greece; 16 Ministry of Rural Development and Food, Minister's Cabinet, 2 Acharnon Str., Athens, Greece; 17 EU-JAMRAI is acknowledged at the end of the article

**Keywords:** antimicrobial resistance, antibiotic, veterinary, animal, surveillance, Europe

## Abstract

Antimicrobial resistance (AMR) should be tackled through a One Health approach, as stated in the World Health Organization Global Action Plan on AMR. We describe the landscape of AMR surveillance in the European Union/European Economic Area (EU/EEA) and underline a gap regarding veterinary medicine. Current AMR surveillance efforts are of limited help to veterinary practitioners and policymakers seeking to improve antimicrobial stewardship in animal health. We propose to establish the European Antimicrobial Resistance Surveillance network in Veterinary medicine (EARS-Vet) to report on the AMR situation, follow AMR trends and detect emerging AMR in selected bacterial pathogens of animals. This information could be useful to advise policymakers, explore efficacy of interventions, support antimicrobial stewardship initiatives, (re-)evaluate marketing authorisations of antimicrobials, generate epidemiological cut-off values, assess risk of zoonotic AMR transmission and evaluate the burden of AMR in animal health. EARS-Vet could be integrated with other AMR monitoring systems in the animal and medical sectors to ensure a One Health approach. Herein, we present a strategy to establish EARS-Vet as a network of national surveillance systems and highlight challenges of data harmonisation and bias. Strong political commitment at national and EU/EEA levels is required for the success of EARS-Vet.

## Background

A One Health surveillance approach is needed to face the challenges of antimicrobial resistance (AMR), as stated in the World Health Organization (WHO) Global Action Plan on AMR [1] and the European Union (EU) One Health Action Plan against AMR [2]. At the EU/European Economic Area (EEA) level, in the human sector, the European Centre for Disease Prevention and Control (ECDC) coordinates the European Antimicrobial Resistance Surveillance Network (EARS-Net), which monitors AMR in invasive bacteria isolated from blood and cerebrospinal fluid in hospitalised patients [3], and the European Food- and Waterborne Diseases and Zoonoses Network (FWD-Net), which monitors AMR in human *Salmonella* and *Campylobacter* infections [4]. In the animal and food sector, the European Food Safety Authority (EFSA) coordinates a mandatory active monitoring of AMR in zoonotic bacteria (*Salmonella* and *Campylobacter*) and indicator bacteria (*Escherichia coli*) from healthy food-producing animals (cattle, poultry, pigs) and food thereof, according to Directive 2003/99/EC [5] and Decision 2013/652/EU [6]. Once a year, ECDC and EFSA produce the EU summary report on AMR in zoonotic and indicator bacteria from humans, animals and food [7]. These coordinated systems highlight significant efforts to produce valuable AMR information at the European level with a public health perspective.

## Veterinary medicine: a current gap in the European surveillance of antimicrobial resistance

Whereas most AMR data in the human sector originate from diseased individuals, the existing surveillance systems lack AMR data from clinical isolates of animals. They are therefore of limited help to assist veterinary practitioners in antibiotic choice and policymakers in regulating veterinary antibiotic use, towards the shared goal of reducing AMR while ensuring optimal treatment of animal infections. Some programmes (VetPath, ComPath and MycoPath), managed by the European Animal Health Study Centre (CEESA, Centre Européen d’Etudes pour la Santé Animale in French) on behalf of a consortium of pharmaceutical companies, produce harmonised AMR data in diseased food-producing and companion animals across EU countries [8], but they cannot replace a continuous European surveillance system for AMR in veterinary medicine.

At the national level, some countries have developed their own surveillance systems for AMR in diseased animals, such as the Czech Republic [9], Denmark [10], Finland [11], France [12], Germany [13], Ireland [14], the Netherlands [15], Norway [16], Sweden [17] and the United Kingdom [18]. However, these systems are fragmented; do not always monitor the same animal species, bacterial species and antimicrobials; and do not always use the same antimicrobial susceptibility testing (AST) methodologies and interpretative criteria. On the other hand, some European countries do not have a surveillance system for AMR in diseased animals in place, even though research-based information may be provided occasionally.

The number of European countries implementing an AMR surveillance system in veterinary medicine is expected to rise and some countries (e.g. Spain) are currently developing surveillance systems, while other countries’ national action plans (e.g. Italy or Belgium) highlight the need and willingness to improve AMR surveillance in this domain. These initiatives present an opportunity to launch coordinated surveillance at the European level and to develop a common surveillance framework to address the lack of harmonisation between national surveillance systems.

## Expected benefits of EARS-Vet

In order to support prudent veterinary use of antimicrobial therapy, and to implement a strong One Health strategy for AMR surveillance and antimicrobial consumption (AMC) control in Europe, building a European Antimicrobial Resistance Surveillance network in Veterinary medicine (EARS-Vet) should be considered. Such a network could be integrated with EARS-Net, FWD-Net, the EFSA monitoring for AMR in zoonotic and indicator bacteria and the European Surveillance of Veterinary Antimicrobial Consumption (ESVAC). This proposal, which we elaborate in the following sections, results from a collective agreement within the EU Joint Action on Antimicrobial Resistance and Healthcare-Associated Infections (EU-JAMRAI). It is in line with Regulation (EU) 2016/429 on transmissible animal diseases (‘Animal Health Law’), which states that *“effective collection and management of surveillance data should be established at Union level […] when relevant, for emerging diseases or antimicrobial-resistant pathogens”* [19], and Regulation (EU) 2019/6 on veterinary medicinal products, which points out the need for AMR data [20].

### Surveillance objectives

EARS-Vet would aim to describe the AMR situation, follow AMR trends and detect emerging AMR in bacterial pathogens of animals in Europe. The data generated could be used for (i) advising policy, (ii) monitoring interventions, (iii) evaluating marketing authorisations of antimicrobials, (iv) supporting antimicrobial stewardship initiatives, (v) generating epidemiological cut-off values, (vi) supporting risk assessment and (vii) estimating antimicrobial resistance burden.

Data generated through EARS-Vet could serve policymakers in devising more targeted and efficient interventions based on a better understanding of the AMR situation and its evolution, as well as of the links between AMC and AMR in animals and humans. At present, these links are investigated in the Joint Inter-Agency Antimicrobial Consumption and Resistance Analysis (JIACRA) reports [21], using AMR data from healthy animals only. Thus, links could be better explored using EARS-Vet data obtained closer to the animals’ point of care, i.e. where antimicrobials are used in veterinary medicine. From 2024 onwards, it will become mandatory for EU countries to provide antimicrobial use data by animal species (in a stepwise approach, starting with the main food-producing animal species) under the framework of EU Regulation 2019/6 [20]. This should make EARS-Vet even more relevant, as resistance patterns in bacterial pathogens may then be linked to antimicrobial use at the animal species level.

EARS-Vet data would also allow for monitoring of the impact of European efforts to tackle AMR in the animal sector, such as the EU One Health Action Plan against AMR and the Animal Health Law [19]. They could also support the evaluation or revision of marketing authorisations of antimicrobials, in the framework of Regulation (EU) 2019/6 [20].

Moreover, EARS-Vet data would support antimicrobial stewardship initiatives, especially the development of veterinary antimicrobial treatment guidelines, which need accurate resistance data of animal pathogens. However, because of differences between countries in terms of animal populations, production systems, bacterial diseases, available authorised antimicrobials and resistance levels, such recommendations would need to be tailored to each country and only general indications could be defined at the European level. In addition, interpretative criteria for AST, i.e. epidemiological cut-off values and clinical breakpoints, are currently missing for many common combinations of animal species, bacterial species and infection sites. This challenges the interpretation of AST results in the veterinary field and from an epidemiological perspective. EARS-Vet could facilitate the defining of missing interpretative criteria by sharing its distributions of AST results to breakpoint-setting organisations such as the Veterinary Committee on Antimicrobial Susceptibility Testing (VetCAST).

EARS-Vet would support assessment of the risk of AMR transmission from animals to humans via non-food-borne routes, e.g. by direct contact with companion or food-producing animals. This would complement the risk assessments performed by EFSA for the food-borne pathway.

Finally, EARS-Vet could provide information to aid in assessing the burden of AMR in animal health, e.g. attributable deaths caused by infections with antibiotic-resistant bacteria. Such an estimation could be similar to the burden assessments performed in the human sector [22], which are particularly useful to raise awareness of the need to tackle AMR.

### Fostering harmonisation and synergies

EARS-Vet would provide the necessary coordination to collectively define common microbiological and epidemiological standards for the surveillance of AMR in animal bacterial pathogens in Europe, as well as a strategy to reach effective harmonisation. More broadly, EARS-Vet would represent an opportunity to build a European scientific community and knowledge hub to support the establishment, improvement and harmonisation of respective national surveillance systems, the interpretation of surveillance outputs and their translation into interventions. This EARS-Vet community could also prove useful in urgent contexts; for example, when an emerging resistance mechanism is discovered and its spread across Europe needs to be quickly evaluated, as experienced in 2015 upon the discovery of plasmid-mediated colistin resistance [23].

## A pragmatic strategy to design EARS-Vet

One of the first steps in designing EARS-Vet would be the definition of a surveillance framework, including its: (i) surveillance scope (i.e. the animal species, production types (e.g. dairy or beef cattle, laying hens or broilers), age categories, bacterial species, specimens and antimicrobials it covers), (ii) AST standards, (iii) metadata to be collected (including minimum and desirable variables), (iv) data governance, (v) frequency of data reporting, (vi) general data management system and (vii) required procedures (including data cleaning and validation), as well as (viii) how data would be analysed and communicated.

This framework should ensure that EARS-Vet can meet the aforementioned surveillance objectives and facilitate broad country participation. It should also address two major challenges for EARS-Vet: the current lack of method harmonisation and possible sampling biases.

To reach harmonisation, we suggest that standards are defined in an inclusive and bottom-up approach, i.e. according to what is considered relevant and feasible within countries. In the beginning, we envisage a transition period where different AST standards would be accepted, as originally done by EARS-Net over two decades, before accepting only those methods complying with the European Committee on Antimicrobial Susceptibility Testing (EUCAST).

The possible sampling biases are linked to the fact that national surveillance systems usually collect AST results routinely produced in veterinary diagnostic laboratories. As culture of veterinary diagnostic specimens is often not performed until treatment failure, AMR levels tend to be overestimated compared with first-time infections [24]. Such biased estimates can have important consequences, including wrongly recommending the use of critically important antimicrobials as first-line treatments when other antimicrobials will actually be effective in the majority of cases. In order to ensure comparability of AMR data between countries, the representativeness of AMR data would need to be assessed before results are interpreted. As in EARS-Net, a series of indicators of national geographic coverage and representativeness could be defined and regularly calculated to understand the validity of the surveillance data. In addition, pragmatic solutions should be explored collectively to address sampling biases. Of note, some countries have decided to subsidise ASTs (e.g. Czech Republic and Spain) to collect more representative AMR data comprising a broader range of cases, i.e. not only those after treatment failure.

## Coordination and sustainability of EARS-Vet

Several successful initiatives, such as EARS-Net and ESVAC, have proven that a European surveillance network can be developed gradually, starting with a group of countries collaborating despite initial lack of harmonisation. Long-term success of EARS-Vet would depend on political commitment at national and European levels, accompanied by sustainable funding. In particular, the European Commission and the EU/EEA countries could play a role in supporting the development of national surveillance systems for AMR in diseased animals and fostering the participation of national agencies in the EARS-Vet initiative.

From a regulatory perspective, the Animal Health Law (applying from April 2021) presents the opportunity to implement AMR surveillance in bacterial pathogens of animals in the EU for the first time. Thus, developing EARS-Vet is timely and could set the basis for a future regulated surveillance network.

## Conclusion

Antimicrobial resistance in animal bacterial pathogens represents a gap in the European One Health strategy on AMR surveillance. Here, we provide a perspective on the multiple anticipated benefits of establishing EARS-Vet and show how such a system could complement the existing European monitoring systems for AMR and AMC, in humans and animals coordinated by ECDC, EFSA and EMA. We have devised a pragmatic strategy to build EARS-Vet, which results from a collective agreement of EU-JAMRAI partners involved in the veterinary and medical sectors. It addresses the two main obstacles that EARS-Vet would face: the current lack of harmonisation in terms of methods and possibly biased surveillance data. In the coming years, more and more countries are expected to build national surveillance systems for AMR in diseased animals. In the absence of a European-level framework, this could lead to a further lack of harmonisation. The Animal Health Law, applying from April 2021, presents the opportunity for possible regulation of AMR surveillance in diseased animals in the EU/EEA, which would coincide well with the establishment of EARS-Vet.
